# Examining the Effectiveness of Naturalistic Social Skills Training in Developing Social Skills and Theory of Mind in Preschoolers with ASD

**DOI:** 10.1007/s10803-017-3377-9

**Published:** 2017-11-20

**Authors:** Grzegorz Szumski, Joanna Smogorzewska, Paweł Grygiel, Ann-Marie Orlando

**Affiliations:** 1 Department of Educational Sciences, The Maria Grzegorzewska University, Szczesliwicka St. 40, 02-353 Warsaw, Poland; 2 Institute of Education, Faculty of Philosophy, Jagiellonian University, Golebia St. 24, 31-007 Cracow, Poland; 3 Department of Psychiatry, Center for Autism and Related Disabilities, University of Florida, Golebia 24 st., PO Box 100234, Gainesville, FL 32609 USA

**Keywords:** Preschoolers with ASD, Social skills, Social skills training, Theory of mind

## Abstract

We compared the effectiveness of two programs for developing social skills, ‘Play Time/Social Time’ (PT/ST) and ‘I Can Problem Solve’ (ICPS), in improving the social skills and theory of mind (ToM) of preschoolers with ASD. The experiment took place in a classroom setting. Fifty-two children attended and data were analyzed with latent growth curve models. Comparison with a control group indicated that both programs were effective in developing social skills. The PT/ST program was more effective than ICPS in developing interaction skills; both programs improved children’s ability to cope with difficult social situations. The ICPS program was marginally effective in developing ToM when compared with PT/ST and control condition. These results are relevant to children with ASD and their teachers.

## Introduction

Social skills and theory of mind (ToM) are two of the most important determinants of social competence in children with autism spectrum disorder (ASD; Baron-Cohen et al. 1985). Unfortunately, children with ASD show delayed development and impairment in both domains (Baron-Cohen et al. 1985). Difficulty with social contact is one of the diagnostic criteria for ASD, and for many years, a delayed ToM was considered the reason why children with ASD developed limited social relationships and was regarded as an essential feature of autism (Baron-Cohen et al. 1985; Leslie and Frith 1988). In recent work on empathizing-systemizing (E–S) theory, Baron-Cohen (2014) emphasized that problems with social contact and communication among persons with ASD are caused by delays in development of empathy. Theory of mind is a cognitive component of empathy, an ability to understand mental states of other people. However, empathy is not only a matter of understanding others’ feelings, but also an ability to react properly in different social situations, which is called ‘emotional empathy’ (Baron-Cohen 2009, 2014; Grove et al. 2014). Therefore, empathy and ToM are often targeted in interventions to improve social skills among children with ASD. Unfortunately, although interventions aimed at developing ToM improve performance on formal ToM tasks they have not been shown to improve real understanding of other people, or to influence the social functioning of people with ASD (Hadwin et al. 1997; Ozonoff and Miller 1995; Swettenham 1996). This might be because programs designed to develop ToM place too much emphasis on the development of ToM *per se*, rather than on applying ToM in real-life social situations. Based on this argument, we investigate whether ToM could be developed using naturalistic programs designed to develop social skills. We assessed the effect of developing ToM in this way on the social functioning of children with ASD in interactive situations. To develop children’s social skills and ToM, we used two well-known programs for developing preschool children’s social competence: ‘I Can Problem Solve’ ICPS (Shure 2000) and ‘Play Time/Social Time’ PT/ST (Odom et al. 1997).

### Social Skills of Preschool-Age Children

Social competence is a very ambiguous concept (Nangle et al. 2010). In recent years, multidimensional hierarchical conceptions have become popular. For example, Vaughn and colleagues (2009) have stated that competent social behavior is based on a constellation of cognitive, behavioral, and emotional factors. Because social competencies are not universal, the capacity to behave appropriately in a variety of social contexts depends, at least in part, on other skills as well as on social competence of a person. Cillessen and Bellmore (2014) argue that social competence should be treated as a two-factor construct, with the first factor being pro-social behavior and cooperation with others and the second relating to the achievement of personal or group goals in social situations. Pro-social behavior requires abilities connected to ToM (i.e., accurate perception of others, the ability to take another person’s perspective, and the ability to understand others’ emotions). Achieving one’s personal aims in social situations requires self-confidence and the ability to argue convincingly and to manipulate other people intelligently. The multidimensional nature of social competence means that it can be measured with several instruments that provide overlapping information about social functioning (McConnell and Odom 1999; Vaughn et al. 2009).

Social skills are very important to children’s development. Children’s social skills usually include showing an interest in others, initiating and sustaining interactions, taking part in group play or goal-directed group activities, responding appropriately to peers’ aggressive behavior, and effectively solving social problem (Jamison et al. 2012; Odom et al. 1999; Van Hecke et al. 2007). These skills are required for positive relationships with peers, which satisfy the need to belong, protect against victimization, and promote cognitive and social development (Miles and Stipek 2006; Parker and Asher 1987). Social skills are also important to the development of children with ASD. There is a large body of evidence that the social isolation of people with ASD is not due to lack of motivation for contact with people, but due to poor social skills (Rumsey et al. 1985; White et al. 2007).

### Theory of Mind

One of the main determinants of competence in social interactions is the ability to take another person’s perspective (Semrud-Clikeman 2007). ToM is the awareness of the thoughts, beliefs, and desires of other people, and is considered a specific aspect of social competence. ToM involves perceiving others as being capable of independent thought and emotion, with their desires and beliefs and the capacity to function independently. ToM also involves an appreciation that a person’s inner state influences his or her behavior (Schaffer 2004; Steerneman et al. 1996). ToM can be considered the cognitive dimension of social skills, as well as the awareness of social rules (Southall and Campbell 2015).

An initial stage of ToM develops until the fifth year of life, emerging from the capacity for symbolic play through understanding of others’ points of view to an eventual understanding of other people’s mental states. Typically developing children display basic skills related to ToM in the fifth year of life (Wellman et al. 2001), but many scholars agree that the beginnings of ToM can be observed in toddlers during the first year of life (c.f., Poulin-Dubois and Chow 2009). Components of ToM typically emerge in the following order: (a) diverse desires (DD)—understanding that people can have different thoughts about the same objects; (b) diverse beliefs (DB)—understanding that people can have different beliefs about the same objects; (c) knowledge access (KA)—understanding that when people do not see something, they do not know about it; (d) false belief (FB)—understanding the false beliefs about hidden objects; and (e) hidden emotion (HE)—understanding that displayed emotions are different from hidden emotions (Wellman and Liu 2004). The development of ToM is delayed in children with ASD, even when compared with children with other developmental delays, and this has given rise to the belief that ToM impairment is the main reason for the difficulties people with ASD have with social interaction (Baron-Cohen et al. 1985).

### Relationship Between ToM and Social Skills

ToM is commonly regarded as a form of social cognition that is responsible for competent social behavior, alongside knowledge of social rules and behavioral norms. At the same time, social skills are a predictor of ToM abilities (Capage and Watson 2001; Slaughter et al. 2002; Werner et al. 2006). The relationship between ToM and competent social behavior is, however, a complex one.

In many cases, young children show many pro-social behaviors, such as helping, comforting, sharing and defending, which are voluntary, intentional, spontaneous, and motivated internally (Grusec et al. 2010). However, pro-socially directed children often are unable to solve tasks designed to assess ToM (Astington 2003; Dunn 1991). It might be that these tasks are too abstract for young children and not emotionally engaging. In addition, it is easy for children to understand people’s emotions and needs when they are easily observed. However, referring to one’s own beliefs and knowledge in contrast with others’ beliefs might be too difficult for young children (Astington 2003; Peskin and Ardino 2003).

In some studies, the converse phenomenon has been observed. That is to say, that the presence of antisocial behaviors in individuals with a well-developed ToM (Sutton et al. 1999) might indicate an advanced understanding of others that can be used as a basis for manipulating and exploiting others for one’s own, egoistic goals (Gasser and Keller 2009; Gini 2006; Heerey et al. 2005).

Social factors such as parenting style, sibling status, social position in the peer group, and individual factors such as temperament and type of attachment between parents and child mediate the relationship between levels of social skills and ToM (Arranz et al. 2002; Carpendale and Lewis 2004; Cassidy et al. 2005; Hoglund et al. 2008; Hughes 2011; Meece and Mize 2010). In summary, although the nature of the relationship is not well understood, it is well established that a relationship exists between social skills and ToM. This is why the simultaneous development of both is regarded as important to children’s development.

### Development of ToM and Social Skills Among Children with ASD

Many interventions have been designed to develop both ToM and social skills in children with ASD; however, evidence on the effectiveness of these interventions remains insufficient (Reichow and Volkmar 2010; Southall and Campbell 2015; White et al. 2007). One of the first systematic reviews of the effectiveness of social skills programs for preschool children with ASD showed that interventions for this group were effective (*d* = 0.66–0.87; Vaughn et al. 2003). However, this analysis covered only two studies in which the relation between intervention and measurement of social skills and accomplished effect was not controlled. While the number of interventions of this type using group designs has increased (Reichow and Volkmar 2010), there is still insufficient evidence to draw firm conclusions about their efficacy. Reichow and Volkmar (2010) concluded that none of the various intervention programs aimed at preschool children with ASD included in their systematic review could be regarded as evidence-based. However, they did point to evidence for the efficacy of other programs for developing social skills, such as those based on Applied Behavior Analysis (ABA), naturalistic techniques, and peer training. An earlier systematic review concluded that only video-modeling interventions met the criteria for an evidence-based practice (Wang and Spillane 2009). The same review concluded that the effects of programs based on social stories and peer-mediated interventions are ambiguous and depend on the method of implementation. Cognitive behavior training was described as a promising technique for developing social skills that has not been studied sufficiently.

The most recent meta-analysis of effectiveness of ToM training, covering 45 studies and over 1500 children, showed that these interventions are fairly effective (Hedges’ *g* = 0.75; Hofmann et al. 2016). This meta-analysis did not, however, analyze the type of disability or method of intervention as moderators. Therefore, it is not clear which ToM training programs or which methods are most effectiveness for children with ASD. A systematic review of various ToM programs for children with ASD concluded that most produced improvements in perspective-taking skills (Southall and Campbell 2015). Unfortunately, the improvements in perspective-taking skills produced by ToM training rarely generalized to real-life situations, and therefore, do not improve children’s ability to interact with their peers or with adults (e.g., Begeer et al. 2011; Ozonoff and Miller 1995; Fisher and Happe 2005).

Many studies of the effectiveness of programs intended to produce improvements in social skills, such as the ability to cope with difficult social situations (Solomon et al. 2004) or conversations (Chin and Bernard-Opitz 2000), found that they did not improve ToM. However, a deeper analysis of these studies included in the review revealed distinct interventions that can improve ToM task-solving and social competencies (Feng et al. 2008; Mackay et al. 2007). These ‘naturalistic’ interventions contained tasks for developing ToM and different aspects of communication, such as listening, conversation, and cooperation skills. However, some flaws, such as a modest number of studies, rather small samples (except for the study of Mackay and colleagues), different ways of ToM assessment and differentiated intensiveness of interventions do not allow inferring about the effectiveness of the interventions for improving ToM and social skills among children with ASD. Further research into the efficacy of naturalistic interventions using a combination of practices is needed to improve understanding of the factors that influence the efficacy of educational interventions and to identify methods for which there is a good evidence base, and can thus be recommended to teachers.

The purpose of this study was to assess the efficacy of two programs designed to develop the social skills of preschool-age children: Play Time/Social Time (PT/ST) and I Can Problem Solve (ICPS). To assess the efficacy of these programs, we used measures of social skills as well as ToM to determine the extent to which the effects of instruction using these programs could be generalized. We observed changes in social skills (i.e., interactional skills and coping with difficult social situations skills) and in theory of mind development in both the experimental and in control groups, as well as correlations between interactional skills, abilities of coping with difficult social situations, and ToM.

Both programs have already been evaluated, but there has not been a comparative study of their efficacy in developing social skills and ToM in children with ASD (Odom et al. 1997; Shure and Spivack 1982; Szumski et al. 2016). Odom and his colleagues improved the structure and content of Play Time/Social Time for a few years to reach the mostly optimal set of lessons and activities (Odom et al. 1997). In a Polish study on effectiveness of PT/ST for social skills development, it turned out that this method was effective for children with different forms of disabilities as well as those without disabilities, showing very strong effect size for both group (*η*^*2*^ = 0.70). The *Time x Group* interaction also was significant with a substantial effect size of *η*^*2*^ = 0.32, indicating that children with disabilities made greater improvement in social skills in comparison to children without disabilities. Children with ASD have shown the smallest changes in comparison to other children, but the differences were marginal (η^2^ = 0.15, *p* = .06). However, there was no control group in this study, which significantly limits its results (Szumski et al. 2016). A study by Shure and Spivack (1982) on effectiveness of ICPS has shown promising results in developing children’s social skills and reducing problems in behaviors. However, the participants in their study were African-American children without disabilities. Thus, it is necessary to determine the effectiveness of this program for children with disabilities.

## Method

### Participants

Fifty-two preschoolers from ten inclusive preschools in Warsaw and its suburbs participated in the study. All children were native Polish speakers. All children had a clinical diagnosis of ASD, were verbal, and had IQ’s above 70. Fourteen children took part in the PT/ST program (10 boys and 4 girls) and 12 participated in the ICPS program (8 boys and 4 girls). The control group of 26 children (18 boys and 8 girls) received no specific training in social skills development other then the standard preschool curriculum. There were no differences between groups in sex distribution (*F*(2, 51) = 0.032; *p* = .97). The mean age of participants was 5,10 years (*SD* = 1.12, range 3,6–7,6). The mean age for each of the three groups was as follows, PT/ST group: 5,4 years (*SD* = 0.83, range 4,6–7,3); ICPS group: 5,0 years (*SD* = 1.2, range 4,0–7,2); control group: 4,9 years (*SD* = 1.2, range: 3,6–7,6). There were no significant group differences for age (*F*(2, 51) = 0.72; *p* = .49.).

We used an adaptation (Pisula et al. 2010) of the Autism Spectrum Quotient: Children’s Version (AQ-Child; Auyeung et al. 2008) to compare the groups for the intensity of core features of autism spectrum disorder. The AQ-Child is a parent-report instrument consisting of 50 items to which responses are given using a four-point Likert scale ranging from ‘definitely agree’ (0) to ‘definitely disagree’ (3). Higher scores indicate the presence of stronger features of ASD. The overall mean score for our sample was 75.10 (*SD* = 16.76). The means for each group were PT/ST group: 76.07 (*SD* = 14.24); ICPS group: 74.72 (*SD* = 21.14); and control group: 74.71 (*SD* = 16.64). The intensity of the features was similar in all three groups (*F*(2, 51) = 0.03; *p* = .97). The mean results approximated the recommended cut-off (Auyeung et al. 2008). However, it is important to highlight that we did not use the measure for diagnosis of ASD and, as the authors stated, the cut-off should be used with caution. Even if our results were slightly lower than the recommended cut-off, all children participated in the study because they had a previous clinical diagnosis of ASD.

### Programs

We used group-based social skills training programs because this method of delivery provides opportunities for children to learn social skills in a naturalistic milieu and promotes interactions with other children (Barry et al. 2003). We chose programs for which there is a manual and formal curriculum because these programs are replicable and easy for teachers to use (White et al. 2007). It is important for future programs implemented in schools to be evaluated and assessed based on their evidence-based and their ease of use for teachers. Most of the programs evaluated previously were delivered by researchers, and it can be difficult for teachers to achieve similar effects (Wang and Spillane 2009). Using a program for which there is a manual and a formal curriculum makes it easier to ensure that the program is delivered in a standardized fashion, in accordance with the manual (McKenna et al. 2014).

Many methods of classifying programs for developing social skills and ToM have been used (Reichow and Volkmar 2010; Vaughn et al. 2003), and consist of a combination of components. Although the programs we chose make use of several components, for this study, the most relevant components of the PT/ST program are the behavioral components so the program can be classified as ABA. The ICPS program relies primarily on cognitive methods.

Play Time/Social Time. PT/ST is aimed at children 3–7 years of age with a disability who have significant problems with social skills development. The PT/ST program is used to teach specific social skills to children of preschool age, starting with the initiation and maintenance of interactions with peers. Specifically, the social skills targeted are: sharing toys, persistence in initiating social interactions, requesting to share objects, play organizing, agreeing with others, and helping other children. The program consists of structured play-based tasks, in which the teacher verbally prompts children to use social skills so that they can have more ‘fun’ interacting with other children. Social interactions in this intervention are not only ‘common play’. During play, the children could talk to each other, exchange materials (e.g., toys), and take turns.

The original version of PT/ST was designed to last 100 days and organized into several phases, during which children perform the activities mentioned above. After 25 social skills sessions, teachers should start to use non-verbal reinforcements (e.g., pictures with ‘Happy Faces’). The reinforcements are mitigated with time—at first the pictures are shown to children when a proper behavior appears, then the pictures are no longer shown to children, but there is a discussion about them at the end of the class, and during the last five sessions also the discussion is discontinued. Each session lasts 15–20 min and is based on a role-play performed in dyads. Each dyad consists of a child with a disability and a child without a disability. Sessions took place every weekday (see also Odom et al. 1997; Szumski et al. 2016).

I Can Problem Solve (ICPS). The program has been designed for children in preschool, kindergarten and elementary school, but the version we were using in the study is devoted to children from 3 to 6 years of age. The aim of the ICPS program is to teach children to solve problems and resolve social conflicts using dialogue. The program consists of 59 sessions, one per day. The program was created for children with special educational needs as well as for typically developing children. Children were grouped in teams with 6–8 children. In the beginning of the program, sessions last no longer than 10 min, and sessions increase in duration with subsequent session lasting 20 min. The sessions consist of play, games, stories, role-playing activities. Children learn how to solve conflicts, to identify their own and others’ emotions, to understand the point of view of another person, and to recognize the reasons for their own behavior and the consequences thereof. As well as giving scenarios for the sessions, the program also includes tips for improving children’s interactions during routine educational situations in preschool.

### Measures

We used three measures in this study. Two of the measures were used to measure social skills and one was used to assess theory of mind development. The measures are described below.

Scholars of social skills agree that these skills should be measured using multiple instruments or instruments with a multidimensional structure (McConnell and Odom 1999). For this reason, we used Polish adaptations (Smogorzewska and Szumski 2015) of two questionnaires that measure slightly different aspects of social skills. The Teacher Impression Scale (TIS; Odom et al. 1997) assesses a child’s ability to enter an interaction and to play with peers. The Taxonomy of Problematic Social Situations questionnaire (ToPSS; Dodge et al. 1985) assesses a child’s ability to cope with difficult social situations and identifies situations, which are especially troublesome for the child.

The Teacher Impression Scale (TIS) was developed by Odom and colleagues (1997). It is a teacher-report instrument consisting of 16 items to which responses are given using a five-point Likert scale, ranging from 1—the child *never* displays this skill, to 5—the child *frequently* displays this skill. The final score is the sum of score on individual items and score are based on observations. Higher scores represent better social skills. The reliability of the scale was high (Cronbach’s *α* = 0.97).

The Taxonomy of Problematic Social Situations for Children (ToPSS; Dodge et al. 1985) is used to measure various aspects of social functioning: peer group entry, response to provocation, response to failure, response to success, social expectations, and teacher expectations. The ToPSS is a teacher-report instrument consisting of 44 items to which responses are given using a five-point Likert scale, ranging from 1—this situation is *never* a problem for the child, to 5—this situation is almost *always* a problem for the child. Lower scores represent better social skills; the lower score the better improvement in child’s behavior. The reliability of the scale was very high (Cronbach’s *α* > 0.97).

Theory of Mind (ToM) was assessed using a scale developed by Wellman and Liu (2004), and revised by Peterson, Wellman, and Liu in 2005, then again by Peterson, Wellman, and Slaughter in 2012. The scale consists of six tasks related to understanding of diverse desires, diverse beliefs, knowledge access, false beliefs, hidden emotions, and sarcasm.

We have added one task, measuring second-order ToM, to the original scale. The ‘Chocolate’ task was developed by Hughes and colleagues (2000) and based on a task designed by Sullivan, Zaitchik, and Tager-Flusberg (1994). The tasks in the scale are ordered from the easiest to the most difficult to prevent a ceiling effect. The instructions for the easiest task in the scale are as follows:

#### Diverse Desire

Here is a lady. This lady wants her morning tea. Here are two foods, a carrot and a biscuit. Pretest Question: Which do you like best? That’s a good choice. But the lady doesn’t like [biscuits]. She likes [carrots]. She loves to eat [carrots] best of all. Test Question: So now the lady can choose only one food. Which will she choose? [If no answer prompt: Will she choose a carrot or a biscuit?] (Correct answer = food the adult likes, always opposite to child’s own preference) (Peterson et al. 2005).

The instructions for the most difficult task in the scale are as follows:

#### Sarcasm

The girl and boy are going on a picnic. It is the boy’s idea. He says it will be a lovely sunny day. But when they get the food out, big storm clouds come. It rains and the food gets all wet. The girl says: ‘‘It’s a lovely day for a picnic.’’ Pretest question: Is it true, what the girl said? Test question: Why did the girl say ‘it’s a lovely day for a picnic? Comprehension control question: Was the girl happy about the rain? (Peterson et al. 2012).

The content of all tasks with the right answers can be found in Peterson et al. 2005 and 2012, and in Hughes et al., 2000.

For this study, we prepared color pictures for all seven tasks to illustrate the stories as they were presented to children. Color pictures of objects (i.e., a carrot, a cake, bushes, a garage) and people (i.e., a girl, a boy, group of children) were shown to the participants. For each task in which the child correctly answered every question, s/he received one point. All seven tasks were assessed in the same way. Children could earn from 0 to 7 points. The correctness of the tasks was assessed by the second author of the article. As there is no room for one’s own interpretation it is not necessary to ask more than one person for independent assessment. The assessment was done in accordance with the original scoring instructions (Hughes et al. 2000; Wellman and Liu 2004; Peterson et al. 2005, 2012). The reliability of the seven tasks is acceptable (i.e., Cronbach’s *α* = 0.80).

We produced Polish translations of the instruments using the back translation technique, translating them into Polish, then once again into English and comparing both English versions to be as similar as possible. Discrepancies between the original and back-translated versions were discussed and resolved. All instruments were validated in a larger sample and demonstrated high reliability; therefore, they were considered suitable for assessing and detecting changes in preschoolers’ skills during the course of the study (Smogorzewska and Szumski 2015).

### Procedure

We conducted the study during the 2014–2015 school year in preschools that had volunteered to take part in the project. Fifty-five preschools from Warsaw and suburbs, which cooperated with us in other, previous projects, received an e-mail detailing the study. To be accepted for the project, the preschool designee had to agree to take part in the study and send permission forms to the children’s caregivers. Twenty preschools from Warsaw and suburbs answered; however, only 10 of the responding preschools had children with ASD in attendance. All parents of children attending the 10 preschools gave permission for their children to participate. Preschools’ groups were assigned randomly to experimental or control groups. Those assigned to the control group were offered the opportunity to follow one of the intervention programs at the end of the study. Forty graduate students who were in the final year of a masters degree in special education and supervised by the children’s preschool teachers delivered the intervention programs. The students follow an inclusive education program at their University, in that during each year of their studies, they had at least 60 h of field practice in inclusive preschools. The preschool teachers supervise the students during their field practice. All supervising teachers graduated from universities. Before the programs began, both teachers and students underwent specific training provided by one of the project leaders, who has experience using the programs with children. She holds a masters degree in education and psychology, and a doctoral degree in education. She teaches college courses on developing social skills and provides social skills trainings. She also has wide experience leading research in the field of social skills development as a principal investigator. The one-day training lasted for five hrs. During the training, the preschool teachers and students learned about the aims of the project and the methods used in program, which the students then implemented. A separate training session was provided for each experimental group. Materials and manuals were provided. At the beginning of the project, teachers assessed the children’s social skills, and doctoral students in special education assessed children’s ToM. Although the teachers were not blind to their assigned study group, they did not choose the program in which they participated. They also did not lead the lessons. We asked them to assess children’s behaviors in different social situations and during different activities, inside and outside the classroom. Also, just after each assessment process we asked them to return the assessment sheets, so that they could not review them and use them for the next assessment process.

To make the duration of the two programs comparable, we shortened the PT/ST program to 67 sessions, cutting some sessions altogether and extending the duration of others from 6 to 10 min. However, we did not omit content from the program, so as not to influence fidelity. In the PT/ST group, children were playing in pairs: child with disability-child without disability. There were up to six pairs playing together at one time. In the case of the ICPS program whole preschool groups participated in each lesson. That is why in the ICPS group more children without disabilities attended the lessons than in the PT/ST group. Lessons were conducted every weekday, Monday to Friday, in the morning or during time, which was mostly suitable for children. Each lesson took no more than 20 min and was part of a daily routine. In the ICPS program manual, suggestions are provided on ways to incorporate the method into the regular preschool program. Lessons took place in the classroom, appropriately prepared for activities (i.e., with needed toys or games). Each lesson was led by one student. There was also a teacher and a teacher assistant in the classroom to help the student as needed. The role of the student was to encourage children to play in pairs with toys they were given and follow the instruction (PT/ST) or to be actively engaged in the class (i.e., playing short games, answering questions, giving ideas, thinking about consequences, etc.; ICPS). The student’s role was also to facilitate children’s play and engagement by giving them verbal and nonverbal reinforcements, but not praising them or telling them the right answer or showing the proper behavior. We reassessed the children’s social skills and ToM at the end of the first phase (i.e., after 28 days in the case of PT/ST; after 33 days in the case of ICPS) and at end of the experiment (after 67 days in the case of PT/ST and 59 days in the case of ICPS).

To monitor the fidelity of program delivery, a random sample of sessions were observed by doctoral students, who completed a specially prepared observation sheet. Its content was prepared in accordance with clues mentioned in Odom et al. (2010) and Harn et al. (2013) with reference to quantity and quality, structure and process of the interventions’ implementation. The observation sheets with Likert scales were used to record the structure of the sessions, duration and course of the sessions, adherence to the planned scenario, leader’s (graduate student) behavior during session (i.e., the way of conducting lesson, giving instructions, reinforcements and support to children), children’s behavior during session (i.e., the motivation to take part in the lesson, reactions to instructions), and interactions between the children and adult. Observers could give a maximum of 16 points in all assessed categories. Higher points on the measure indicated better fidelity and meant better accordance with programs’ instruction, structure and content. The results of the observation were always compared with the instruction and content of the concrete lesson. For each experimental group, 12 observations were completed. An analysis of variance (ANOVA) showed that overall, the sessions conducted during the study were consistent with the programs’ assumptions, and there were no statistical differences between groups (see Table 1). The given data indicate that the fidelity was high and close to maximum. Analyses show that such a level of fidelity is acceptable and should not negatively affect the effectiveness of the intervention (Durlak and DuPre 2008). Moreover, it is worth noting that teachers who also attended the training upon implementing the programs constantly supervised students who led the classes with preschoolers. It could be a reason for the high level of fidelity. However, if the observer saw any incorrect procedures during the session, there was always a brief discussion afterwards, during which observer as well as leader could express their thoughts and doubts. In such case the observation was repeated to check whether the quality of the lesson changed for the better. This observation, though, was not included in the fidelity data. It is also important to note that in all groups the programs were completed and we did not observe any significant changes in the programs’ implementations in time.

**Table 1:** Fidelity

	Play time/social time				I can problem solve				ANOVA
	*M*	*SD*	*Min*	*Max*	*M*	*SD*	*Min*	*Max*	
The structure	3.83	0.39	3	4	3.91	0.29	3	4	*F*(1, 23) = 0.36, *p* = .55
Teacher’s behavior	5.08	0.66	4	6	5.42	0.66	4	6	*F*(1, 23) = 1.49, *p* = .24
Children’s behavior	5.25	0.87	4	6	5.25	0.96	4	6	*F*(1, 23) = 0, *p* = 1.0
Overall	14.17	1.27	12	16	14.58	1.68	12	16	*F*(1, 23) = 0.47, *p* = .50

### Statistical Analysis

To determine if ToM correlated positively with the interactional skills of children with ASD, we used Pearson’s product-moment correlation coefficient. To compare the rates at which with ASD assigned to either the experimental and control groups developed interactional skills, coping skills, or ToM, we employed a series of latent growth curve models (LGCMs; Bollen and Curran 2006; Duncan and Duncan 2004; McArdle and Epstein 1987). LGCMs are one of the main ways of analyzing level and change in longitudinal data. They provide information about the growth or decline in variables at the participant level by estimating an underlying temporal trajectory for each individual.

The basic idea behind these models is that the change in a participant’s scores on the measures (i.e., growth or decline) on a given variable is a function of a latent random intercept (i.e., the average initial value at the start of the longitudinal change process) and a latent random slope (i.e., the average individual rate of change over time). LGCMs also allow for estimation of the variance in the intercept (i.e., the individual variability around the group baseline parameter) and slope (i.e., the individual variability around the group change parameter). Statistically significant variance indicates that participant trajectories of change do not follow the same pattern.

An important advantage of LGCMs is that they enable the study of predictors of change in a participant’s scores over time (i.e., variance in growth processes between subgroups can be examined). In other words, LGCMs can be used to investigate whether, and to what extent, predictors can account for variance at baseline and change over time. In this study, we evaluated intervention type (i.e., PT/ST, ICPS, control) as a predictor of both slope and intercept constructs (i.e., TIS, ToPSS, ToM). A dummy code approach was used to compare the impact of PT/ST (dummy code = 1), ICPS (dummy code = 1) and the control condition (i.e., reference group; dummy code = 0) on intercept and slope dependent variables.

**Fig. 1:**
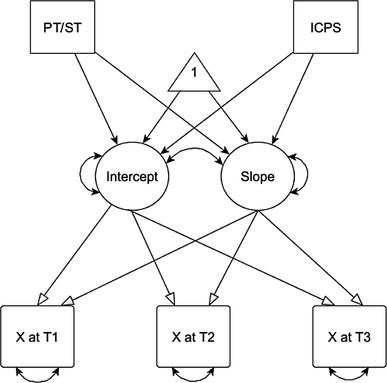
Conditional univariate latent curve model. *Note PT/ST* play time/social time, *ICPS* i can problem solve, *Intercept* mean initial status, *Slope* mean rate of change

As an initial analysis, we ran separate unconditional models (i.e., models without covariates) to describe the baseline values and rates of change of the three constructs under investigation (i.e., TIS, ToPSS, ToM). We then examined the associations between baseline values and rates of change in TIS, ToPSS and ToM. After this, we investigated whether we could predict the baseline values and rates of change by running conditional models including predictors such as intervention type (see Fig. 1).

Statistical analyses were performed using Mplus 7.4 (Muthén and Muthén 2012) and Bayesian estimator (Van de Schoot et al. 2014). We used Bayesian statistics because they perform well in small samples and are robust against non-normality in the data (Van de Schoot et al. 2015), but this meant that well-known fit indices (e.g., chi-squared, RMSEA, CFI, TLI) were not available (Byrne 2011; Geiser 2013). We therefore used the posterior predictive p-value (PPPV) as an index of goodness of fit. Small, significant (*p* < .05) values of PPPV indicate poor model fit. In specifying the linear growth model, we constrained the factor loadings of the intercept to 1 and the factor loadings of the slope to 0, 1 and 2 (time-lags between assessments).

To provide appropriate effect sizes, differences in rate of change (slope) between conditions (experiment vs. control, dummy-coded) were estimated using StdY standardization parameters. These coefficients express the expected change in standard deviations of the dependent variable (a random slope) when the independent variable (experiment vs. control) changes from zero to one. Because the independent variables are all coded as dummy variables, the coefficients are directly comparable as they indicate the relative impact of the related independent variable. Therefore, these effect sizes can be interpreted as similar to Cohen’s *d*.

## Results

### Preliminary Analyses (Correlations and Unconditional LGCM)

Correlation matrix between variables. The data in Table 2 show that TIS scores were not correlated with ToPSS scores in any of the assessments (see the intersection of columns 2–4 and rows 5–7). TIS scores and ToM, however, were correlated in all the assessments (*r* = ~ 0.6) (see the intersection of columns 2–4 and rows 8–10). Thus, ToM correlates positively with the interactional skills of children with ASD.

**Table 2:** Means, standard deviations, and correlations

Variable	*M*	*SD*	1	2	3	4	5	6	7	8	9
1. AQ	75.10	16.76									
2. TIS 1	44.79	13.65	− 0.33*								
3. TIS 2	48.14	14.12	− 0.39**	0.84**							
4. TIS 3	52.29	14.37	− 0.35*	0.78**	0.86**						
5. ToPSS 1	2.91	0.78	− 0.08	− 0.12	− 0.08	0.01					
6. ToPSS 2	2.76	0.73	− 0.05	− 0.00	− 0.04	0.10	0.80**				
7. ToPSS 3	2.64	0.69	0.02	− 0.10	− 0.14	− 0.18	0.74**	0.86**			
8. ToM 1	2.17	1.83	− 0.02	0.58**	0.57**	0.56**	− 0.10	0.08	− 0.06		
9. ToM 2	2.38	1.72	− 0.19	0.54**	0.59**	0.54**	0.06	0.15	0.01	0.79**	
10. ToM 3	2.76	1.83	0.02	0.51**	0.56**	0.56**	− 0.03	0.18	− 0.04	0.76**	0.76**

AQ autism quotient, TIS Teacher Impression Scale, *ToPSS* taxonomy of problematic social situations for children, *ToM* theory of mind. TIS 1, 2, 3, ToPSS 1, 2, 3, ToM 1, 2, 3—the first, second and third assessment

*indicates *p* < .05; ** indicates *p* < .01. *M* and *SD* are used to represent mean and standard deviation, respectively

Latent growth curves without covariates (unconditional models). We first calculated separate latent growth curves without covariates to describe the baseline and rate of change in TIS, ToPSS and ToM.

### LGCM for TIS

The PPPV for TIS was not significant (*p* = .548), indicating that the linear growth curve described the data accurately (see Table 3). The means for the baseline and average rate of change (see Table 3) were 44.64 and 3.77 (*SD* = 0.70, *p* < .001) respectively, indicating that TIS scores increased over time. Analysis of the variance in baseline and rate of change revealed differences between individuals with respect to both baseline and average rate of change (184, *p* < .001), which justified the addition of variables that might account for individual variation in trajectory. The TIS intercept and change factors were uncorrelated (*p* > .05).

**Table 3:** Unconditional latent growth curve Model for TIS, TOM and TOPSS

	TIS		ToPSS		ToM	
	Estimate (SD)	95% CI	Estimate (SD)	95% CI	Estimate (SD)	95% CI
Mean initial status	44.64 (2.02)***	[40.66, 48.63]	2.90 (0.11)***	[2.67, 3.12]	2.16 (0.27)***	[1.62, 2.70]
Mean rate of change	3.77 (0.70)***	[2.33, 5.14]	− 0.13 (0.04)**	[− 0.21, − 0.05]	0.30 (0.11)**	[0.09, 0.53]
Initial status variance	184.02 (46.52)***	[115.84, 296.82]	0.57 (0.15)***	[0.35, 0.93]	3.08 (0.86)***	[1.82, 5.15]
Rate of change variance	12.74 (7.32)***	[1.33, 28.68]	0.04 (0.02)***	[0.01, 0.09]	0.15 (0.12)***	[0.01, 0.47]
Correlation between initial status and rate of change	− 6.51 (12.77)	[− 34.07, 16.26]	− 0.06 (0.04)	[− 0.16, 0.01]	− 0.23 (0.27)	[− 0.83, 0.22]

TIS Teacher Impression Scale, *ToPSS* taxonomy of problematic social situations for children, *ToM* theory of mind. Parameters are unstandardized

***p < .001, **p < .01

### LGCM for ToPSS

The unconditional LGCM for ToPSS was also an excellent fit to the data (PPPV *p* = .53). The mean of the intercept or baseline for ToPSS was 2.90 (*p* < .001) and the variance was 0.57 (*p* < .001). The mean for the estimated slope of ToPSS was − 0.13 (different from zero: *p* < .01), indicating that the average ToPSS score decreased over time. There also was individual variance in the slope parameter (0.04, *p* < .001), indicating that the rate of change in ToPSS varied amongst the children. The covariance between the latent intercept and slope was negative, but non-significant (− .06, *p* > .05).

### LGCM for ToM

As in the case of the other dependent variables, the unconditional LGCM for ToM was a good fit to the data (PPPV *p* = .36). The mean baseline value for ToM was different from 0 (2.16, *p* < .0001) and varied between participants (3.08, *p* < .001). There was an increase in ToM over time (mean rate of change = 0.30, *p* = .01) that varied between participants (0.15, *p* < .001). As in the TIS and ToPSS models the baseline value for ToM was not related to the rate of change (*p* > .05).

### Main Analysis (Conditional LGCM)

Because the main aim of the study was to assess whether the PT/ST and ICPS programs were effective in improving children’s social skills and ToM, the next step in the analysis was the estimation of LGCMs in which being in the intervention group was a predictor of: (a) baseline value, and (b) change in all the dependent variables (TIS; ToPSS; ToM).

#### LGCM for TIS

We start by describing the analysis of the TIS model, which was a good fit to the data (PPPV = 0.15, see Table 4). Table 4 shows that at pre-test, TIS scores were similar in the experimental groups and the control group. In the case of PT/ST and ICPS, the regression coefficient for initial status of TIS was insignificant (*p*s > 0.05). The rate of change in TIS was faster in the PT/ST group than in the control group, indicating that the PT/ST program accelerates the development of abilities measured by TIS (*β*_*STDY*_ = 1.48, *p* < .01). The ICPS group showed a similar rate of change in TIS to the control group (*β*_*STDY*_ = 0.63, *p* > .05). In fact the PT/ST program was more effective than the ICPS program, which did not improve the aspects of social skills captured by the TIS.

**Table 4:** Results for the conditional models predicting the initial status and rate of change for TIS, TOM and TOPSS

	TIS		ToPSS		ToM	
	Estimate (SD)	95% CI	Estimate (SD)	95% CI	Estimate (SD)	95% CI
Initial status regressed on						
← PT/ST	− 0.30 (0.35)	[− 0.94, 0.40]	0.83 (0.30)**	[0.18, 1.35]	0.03 (0.34)	[− 0.63, 0.69]
← ICPS	− 0.43 (0.35)	[− 1.07, 0.30]	1.20 (0.29)***	[0.55, 1.67]	− 0.57 (0.36)	[− 1.22, 0.19]
Rate of change regressed on						
← PT/ST	1.48 (0.41)**	[0.62, 2.22]	− 1.65 (0.35)***	[− 2.20, − 0.84]	0.21 (0.57)	[− 0.97, 1.34]
← ICPS	0.63 (0.43)	[− 0.23, 1.47]	− 1.18 (0.39)**	[− 1.87, − 0.36]	0.94 (0.63)	[− 0.35, 2.15]
Initial status intercept	47.07 (2.90)***	[41.36, 52.79]	2.52 (0.14)***	[2.23, 2.80]	2.36 (0.38)***	[1.62, 3.11]
Rate of change intercept	1.79 (0.90)*	[0.02, 3.54]	0.02 (0.05)	[− 0.07, 0.12]	0.20 (0.15)	[− 0.10, 0.49]
Initial status variance	181.53 (48.07)***	[112.94, 301.05]	0.42 (0.12)***	[0.24, 0.71]	3.00 (0.85)***	[1.74, 5.08]
Rate of change variance	8.34 (5.57)***	[0.84, 21.66]	0.02 (0.01)***	[0.01, 0.06]	0.17 (0.13)***	[0.02, 0.51]
Correlation between initial status and rate of change	− 0.03 (0.31)	[− 0.52, 0.73]	− 0.04 (0.34)	[− 0.58, 0.77]	− 0.26 (0.37)	[− 0.86, 0.66]

TIS Teacher’s Impression Scale, *ToPSS* taxonomy of problematic social situations for children, *ToM* theory of mind, *PT/ST* play time/social time, *ICPS* i can problem solve. Regression and correlation coefficients are standardized (STDY for regression; STDYX for correlation). All others paramteres are unstandardized

***p < .001, **p < .01, *p < .05

**Fig. 2:**
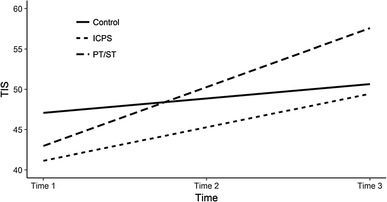
Estimated growth curves for Teacher’s Impression Scale (TIS) over time. *Note TIS* Teacher’s Impression Scale, *Control* control group, *PT/ST* play time/social time, *ICPS* i can problem solve

It is worth noting that adding predictors of pre-tests and change to the model did not lead to homogenization of variance in either baseline or rate of change. Both coefficients were still statistically significant (*p* < .001). In the case of TIS, the interventions are not sufficient to account for the variance in trajectories (Fig. 2).

#### LGCM for ToPSS

**Fig. 3:**
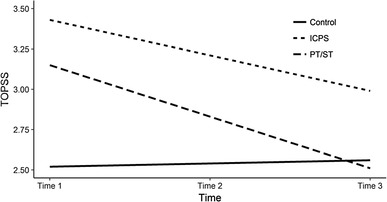
Estimated growth curves for taxonomy of problematic social situations for children (TOPSS) over time. *Note ToPSS* taxonomy of problematic social situations for children, *Control* control group, *PT/ST* play time/social time, *ICPS* i can problem solve

We observed clearer situations for the ToPSS model (PPPV = 0.19; see Table 4). First of all, both intervention groups had higher (i.e., worse) pre-test ToPSS scores than the control group (see Table 4; Fig. 3; PT/ST: *β*_*STDY*_ = 0.83, *p* < .01; ICPS: *β*_*STDY*_ = 1.20, *p* < .001). Both intervention groups showed a greater decrease in ToPSS scores over time than the control group (PT/ST: *β*_*STDY*_ = − 1.65, *p* < .001; ICPS: *β*_*STDY*_ = − 1.18, *p* < .001). In other words, in both groups the reduction in inappropriate behaviors during the course of the experiment was larger than in the control group.

In this context, it is important to note that when intervention type was used as a predictor of change in ToPSS the rate of change of intercepts was not significant, indicating that taking part in the intervention decreased problematic behaviors among children. At the same time, the variance in pre-test for the ToPSS and in rate of change of ToPSS remained significant (*p* < .001), which indicates that the intervention variable was not sufficient to account for pre-test differences in ToPSS or the changes in ToPSS during the course of the experiment.

#### LGCM for ToM

**Fig. 4:**
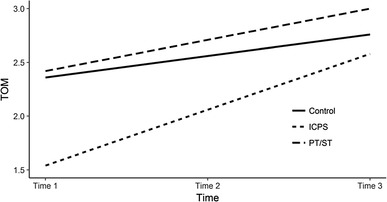
Estimated growth curves for theory of mind (TOM) over time. *Note ToM* theory of mind, *Control* control group, *PT/ST* play time/social time, *ICPS* i can problem solve

There were no group differences in ToM in the pre-test (see Table 4; Fig. 4). In the case of PT/ST and ICPS the regression coefficient for baseline was not significant (*p* > .05). There was also no difference between the PT/ST and control groups with respect to rate of change in ToM (*β*_*STDY*_ = 0.21, *p* = .35). It is less clear whether the ICPS program had any influence on ToM development (*β*_*STDY*_ = 0.94, *p* = .07). The regression coefficient ICPS→ToM_RateOfChange_ was not different from zero using the standard *p* < .05 criterion for statistical significance; however, if we use a more liberal criterion (*p* < .1), we cannot reject the possibility that the ICPS improved ToM development. Marginal effectiveness of the ICPS program indicates a 95% confident interval (CI) of estimated coefficient, which only insignificantly includes 0 [− 0.35, 2.15]. A positive regression coefficient would indicate faster positive changes in ToM in the ICPS group in comparison with the control group.

To determine whether or not the calculated *p* value was caused by a small number of participants (Hoyle 1999), a post-hoc power analysis was performed based on Monte Carlo simulation (Muthén and Muthén 2002). With a sample size of 51 and the results of the study as population values with 100,000 replications, a simulation indicated that given the observed magnitude of interesting coefficient, the sample size, and α = 0.05, the calculated power was 0.94. Following Cohen (1988) statistical power estimates (i.e., 0.8 and above), this means that, under these conditions, there was a 94% chance that this coefficient would be proven to be statistically significant (it should be statistically insignificant). This result reaffirms that the *p* value of regression coefficient obtained in our analysis emerges from a small sample. Obviously, replicating the results on a larger sample is needed to confirm the conclusions we have reached.

An indirect argument, showing the effectiveness of ICPS method, is that the intervention variable in the model causes that the ToM intercept rate of change is statistically insignificant. In other words, if we had not conducted the interventions (i.e., all predictors had a 0 value), there would have been no change in the children’s ToM level. The results indicate that the ICPS intervention was relatively more effective than the PT/ST.

As in the case of the other dependent variables, the intervention variable did not completely account for pre-test differences in ToM or the changes observed during the course of the experiment. Both parameters remain statistically significant (*p* < .001).

## Discussion

We assessed whether two different intervention programs were more effective than the standard preschool curriculum in developing social skills (i.e., interactions with peers, coping with difficult social situations) and ToM in preschool children with ASD. The given results are discussed with reference to three main issues: the relation between two aspects of social skills and ToM in the examined groups, the effectiveness of two programs in developing social skills and the effectiveness of these programs in developing ToM.

We showed that ToM is correlated with the ability to initiate contact with peers. ToM and TIS scores were highly correlated in each of the measurement times as well as between the measurement times. These results corroborate previous research showing that ToM is important for establishing and maintaining positive relations with others, because an understanding of other people and their thoughts, beliefs and behaviors is what makes an individual capable of reacting and responding to others (Astington 2003; Cutting and Dunn 2006; Davies and Stone 2003; Howe et al. 2002).

At the same time, we found that children’s ToM development was not related to the frequency with which they displayed problematic social behaviors. In other words, having a more developed ToM does not prevent difficulties in social contact with peers, and a poorly developed ToM is not a predictor of problematic social behavior. A previous study on the relationship between ToM development and preschoolers’ ability to cope with solving difficult social situations produced ambiguous results (Capage and Watson 2001). Although such a relationship was observed, it is weak and disappears after controlling for some variables (e.g., age and language skills) (Capage and Watson 2001).

Not all difficult social situations relate to interactions; some situations concern adjustment to social norms and fulfillment of standard social roles, such as being a student (Dodge et al. 1985; Servik et al. 2010). ToM, however, evinces mostly in interpersonal situations and in interactions important for both partners, which require understanding of the partner’s thoughts, emotions, and goals. However, some interpersonal situations have negative contexts (e.g., a peer’s provocation). The ability to regulate one’s emotions, self-efficacy with respect to contacts with others and the ability to generate many solutions are much more important factors in coping with this kind of situation than insight into others’ thoughts and feelings (Dodge et al. 1985). Although the relationship between ToM and social skills was not a main subject of this study, the results are important for better understanding of ToM significance for social functioning of children with ASD. They support a well-established belief that the level of ToM development is strongly connected with interaction skills, but it is not important for ability to cope with difficult social situations.

Two conclusions can be drawn from our comparison of the effectiveness of two programs in developing the social skills of children with ASD. First, it is worth emphasizing that we found evidence that the programs produced positive improvements in children with ASD, even though it is often difficult to achieve positive results with social developmental interventions in this population (Hadwin et al. 2015; Southall and Campbell 2015; Vaughn et al. 2003).

Second, the PT/ST program, based on behavioral methods and peer interactions in natural settings, produces more general effects than ICPS—a cognitive program. The ICPS program only improved social problem solving, whereas the PT/ST program influenced both interaction skills and social problem solving, which makes it more effective. Our results are in line with those from previous studies demonstrating that behavioral interventions based on naturalistic techniques and peer training are amongst the most effective methods of improving social skills of children with ASD (see: Reichow and Volkmar 2010). For children with ASD, those taking part in the PT/ST program interaction skills improved almost one-and-a half standard deviations more than the control group (*β*_*STDY*_ = 1.48, *p* < .01). Also, problems in coping with difficult social situations decreased over one-and-a half standard deviations (*β*_*STDY*_ = − 1.65). The changes are strong, with visible clinical importance. However, it is not easy to compare them directly to effects received in most previous interventions focused on improving social skills among children with ASD. The reason for this is that most of the studies were single-case research (Bellini et al. 2007; Reichow and Volkmar 2010), for which quantitative methods of effect size assessment are in progress and are incomparable with measures used in studies of bigger groups. Results of meta-analysis of single-case studies highlight that effects of programs developing social skills among preschool children with ASD are moderate (Bellini et al. 2007). Rare results of group studies are more promising and show strong (Kasari et al. 2006) and very strong effects (Kroeger et al. 2007; Smith et al. 2004). Comparing effects of different studies demands a lot of caution, because these studies represent different ways of understanding social skills and different ways of assessing them (Rao et al. 2008).

The effectiveness of the ICPS program provides support for the idea that cognitive programs can improve at least some aspects of social competence in children with ASD (Wang and Spillane 2009). Problems in coping with difficult social situations among children taking part in the ICPS program decreased one standard deviation (*β*_*STDY*_ = − 1.18, *p* < .001); however, there is no change in the case of interaction skills. The ICPS program thus brings less generalized improvement of social skills than the PT/ST program.

Unlike the PT/ST program, the ICPS program showed promise as a method of developing ToM in children with ASD. Analysis indicated that the ICPS program produced a marginal improvement (*p* < .1) in ToM, and modeling indicated that had the ICPS group not been included in the sample, the overall increase in ToM level would have been much smaller. Moreover, an additional statistical simulation shows that the *p* value in this case is caused by an objectively small sample size. This result requires some discussion.

First, results of the current study, therefore, are consistent with results of Bauminger (2007) and Bauminger-Zviely et al. (2013) and support a thesis that some social skill interventions bring more general results and improve ToM. Replication of this positive result with other social skills programs can influence the quality of education of children with ASD. Positive results might also increase ToM development (Begeer et al. 2011; Fisher and Happe 2005; Ozonoff and Miller 1995).

Second, our study shows that cognitive programs can be more effective in developing ToM than behavioral programs, which is logical because ToM is a cognitive ability, in contrast to the behavioral aspects of social competences (Langeveld et al. 2012). Our study provides support for the notion that there are two independent aspects of social competence whose development depends on different experiences.

The focus of the ICPS is on teaching children how to resolve conflicts with their peers. Resolving conflicts in a way that satisfies all the parties requires insight into the needs and wishes of the other parties, as well as one’s own (Johnson and Johnson 1996). The ICPS program makes extensive use of peer group discussion, which creates plenty of opportunities for exposure to other people’s arguments. It is also not unimportant that the ICPS program includes language activities, which might help to develop participating children’s language skills. There is some evidence that children’s ability to solve ToM tasks is correlated with their linguistic competence (Milligan et al. 2007).

It is worth noting that the ICPS program, like the PT/ST program, was not designed specifically for use with children with ASD. Both programs are intended to be use with inclusive groups consisting of a mixture of children with and without special educational needs. They can be used in natural settings in preschools (i.e., be a part of the everyday preschool routine), which is a great advantage (Rao et al. 2008). There is growing demand for inclusive programs of this type because there is systematic growth in the number of children diagnosed with ASD (Goodman and Williams 2007), as well as growth in their participation in inclusive education contexts (Loiacono and Valenti 2010). Many teachers do not feel prepared for work with children who have ASD and the availability of programs with a formal curriculum that is manualized might improve teachers’ ability to meet the needs of children with ASD (White et al. 2007).

### Limitations

Before concluding, a few potential limitations of our study should be mentioned. One of them is the rather small sample. Additionally, the participants were assessed only with the AQ questionnaire; we did not use other, more comprehensive instruments, such as the ADOS-2 or ADI-R. However, children had a previous clinical diagnosis of ASD and we used AQ only to compare the intensity of autism features in the experimental and control groups. Statistical analysis showed that the groups were comparable in this case.

Although the children taking part in the study were verbal, we did not assess the preschoolers’ receptive and expressive language or their communication skills. Therefore, we do not know whether or not potential problems in communication lead to weaker results in effectiveness among individual children. This could impact the effectiveness for the ICPS program in particular as children communicate verbally with the group leader. Limited communication skills can reduce potential effectiveness of the method. The role of language abilities in methods’ effectiveness should be considered in the next study.

Due to small number of children within each group and larger number of boys than girls, we were not able to determine differences in the effectiveness of the methods in the case of sex. Although, this factor is worth checking in the next analysis, there are no theoretical premises to hypothesize that the programs affect girls’ and boys’ development differently.

An important limitation is that assessments of social skills were not blind. The teachers who assessed the children were aware that they were taking part in the experiment although they were not key players in delivery of the intervention. Delivery of the intervention was led by graduate students, as part of their internship.

The most important weakness of our study is the lack of a formal follow-up. We do not know whether the improvements we observed were enduring, nor whether they led to further positive changes in participants’ behavior and abilities. Such information is very important in determining the practical value of the programs, as well as from a theoretical perspective. Moreover, there is a lack of measurement of generalization of the skills learned. We do not know whether participation in the project positively changed children’s everyday functioning in social situations other than preschool. This issue needs considering during further studies.

## Conclusions

In our study, despite some limitations, we were able to show that the Play Time/Social Time (PT/ST) and I Can Problem Solve (ICPS) interventions are more effective than the standard preschool curriculum in developing interactional skills and in teaching kids how to cope with difficult social situations. The PT/ST program is more effective than ICPS in developing interactional skills; however, both programs are similarly effective in teaching how to cope with difficult social situations. In contrast, only the ICPS program develops ToM. Our results are clinically as well as practically important. They show that it is possible to improve social functioning of children with ASD, as well as their theory of mind using educational programs.
